# Metabolomics-based biomarker discovery for bee health monitoring: A proof of concept study concerning nutritional stress in *Bombus terrestris*

**DOI:** 10.1038/s41598-019-47896-w

**Published:** 2019-08-06

**Authors:** Luoluo Wang, Ivan Meeus, Caroline Rombouts, Lieven Van Meulebroek, Lynn Vanhaecke, Guy Smagghe

**Affiliations:** 1 Department of Plants and Crops, Faculty of Bioscience Engineering, Ghent University, Ghent, Belgium; 2 Laboratory of Chemical Analysis, Department of Veterinary Public Health and Food Safety, Ghent University, Ghent, Belgium

**Keywords:** Entomology, Metabolomics, Environmental impact, Entomology, Environmental impact

## Abstract

Bee pollinators are exposed to multiple natural and anthropogenic stressors. Understanding the effects of a single stressor in the complex environmental context of antagonistic/synergistic interactions is critical to pollinator monitoring and may serve as early warning system before a pollination crisis. This study aimed to methodically improve the diagnosis of bee stressors using a simultaneous untargeted and targeted metabolomics-based approach. Analysis of 84 *Bombus terrestris* hemolymph samples found 8 metabolites retained as potential biomarkers that showed excellent discrimination for nutritional stress. In parallel, 8 significantly altered metabolites, as revealed by targeted profiling, were also assigned as candidate biomarkers. Furthermore, machine learning algorithms were applied to the above-described two biomarker sets, whereby the untargeted eight components showed the best classification performance with sensitivity and specificity up to 99% and 100%, respectively. Based on pathway and biochemistry analysis, we propose that gluconeogenesis contributed significantly to blood sugar stability in bumblebees maintained on a low carbohydrate diet. Taken together, this study demonstrates that metabolomics-based biomarker discovery holds promising potential for improving bee health monitoring and to identify stressor related to energy intake and other environmental stressors.

## Introduction

Bees are perhaps the best known beneficial insects, performing ecosystem services which are vital to both food security and biodiversity^1^. More specifically, bumble bees are key pollinators in temperate climate regions^2^, and the economic value derived from their pollination services is worth billions of dollars annually^3^. However, bee diversity and their essential pollination services are threatened^4,5^. Understanding synergistic/antagonistic interactions between drivers of decline, to ultimately identify dangerous combined effects, will be critical to save bees^6,7^. Having robust tools to measure bee health would allow us to identify how health relates to biotic and abiotic stressors. Meanwhile, pollinators, especially honey bees, have been employed as biological indicators to monitor environmental pollution since 1962^8^, as well as to identify diseases and parasites in relation to chemical and physical factors^9,10^. Although relations with environmental stressors can be drawn, no identification or quantification on the severity of a specific stressor on the bees can be inferred. Hence, given that the biomarker-approach is capable of linking the physiological status of bees to the severity of specific stressor, the development of an effective and practical approach, which can identify the diagnostic biomarkers tracking a single stressor and its interplay with others, is crucially needed.

In this context, metabolomics has been shown to have some advantages over other post-genomic technology. The metabolome is the final downstream product of gene transcription, and therefore, it is the closest to the phenotype of the biological system studied^11^. Additionally, unlike the transcriptome or the proteome which are diversified from species to species, the basic metabolic pathways and their metabolites among different species are the same, making metabolomic analysis much more universal^12^. Moreover, metabolomic approaches have been successfully developed for environmental relevant species for biomarker discovery and risk assessment of toxicant exposure, metabolic responses to environmental stressors, and disease diagnosis and monitoring^13,14^. Within the field of entomology, the metabolomic approach was first applied in 1990 with the evaluation of the metabolism of parasitized *Manduca sexta* larvae^15^. Since then, metabolomics has been applied to a wide range of insect research including honey bees, providing new insights into biological processes that could hardly be obtained using any other approach^16,17^. Hence, this study has the objective to perform mass spectrometry-based metabolomics to identify biomarkers that can discriminate bee health status with or without an introduced single stressor.

Bees depend entirely on nutrition obtained through floral pollen and nectar for growth, reproduction, and health^18^. Growing evidence has linked the interaction between malnutrition and other stressors with current bee population declines^18–20^. Although this is not yet fully understood, it seems highly likely that nutritional stress is significantly contributing to the synergistic effect of numerous other stressors^18,20^. For instance, poor nutrition due to loss of food sources could be synergistically acting with emerging pathogens to cause bee population decline^20,21^. Moreover, some pathogens are known to directly affect the energy metabolism in bees^22,23^, but not always^24^. Hence, tools to identify in which environments bees suffer nutritional stress are therefore key for good conservation planning. Following this, we setup this proof-of-concept study, with the focus on testing the ability of a metabolomics-based approach to classify nutritional status of bees, whereby a mimic of low carbohydrate food forage stress was imposed to *Bombus terrestris*, a key pollinator in temperate climate regions of Europe^25^.

## Results and Discussion

**Figure 1:**
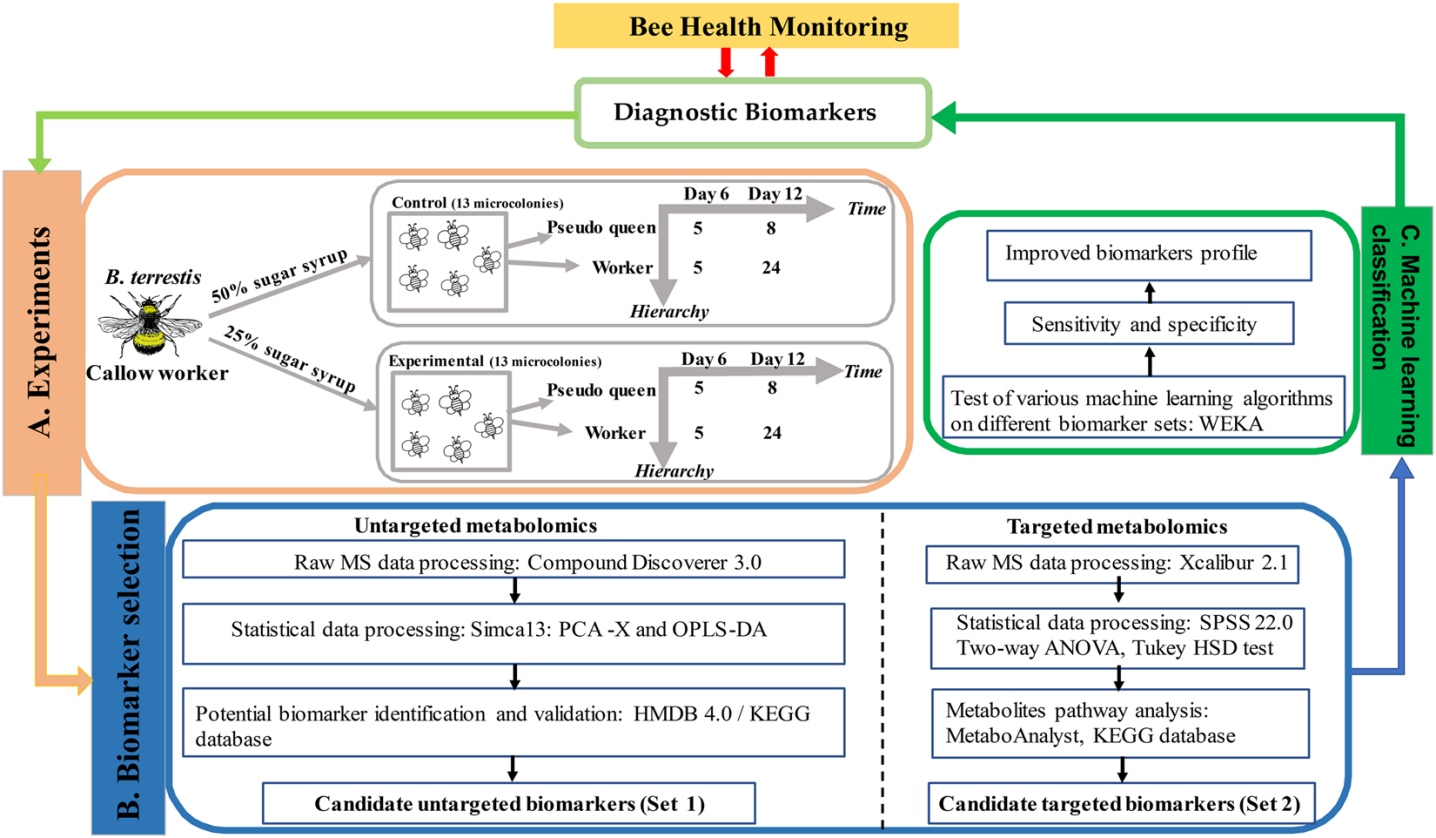
Schematic diagram of the metabolomics-based bee health monitoring workflow. (**A**) Experimental setup in this prove-of-concept study with commercial bees and diluted sugar syrup diet. Five random *Bombus terrestris* callow workers were allocated to one microcolony. A total of 26 microcolonies were randomly assigned to the treatment (25% sugar syrup, 13 microcolonies) or control (50% sugar syrup, 13 microcolonies) groups. We sampled 2 specimens at day 6 (in 2 × 5 microcolonies) and 4 specimens at day 12 (in the other 2 × 8 microcolonies). In total for day 6 this is 10 dominant pseudo queens and 10 workers, for day 12 this is 16 dominant pseudo queens and 48 workers. Collected bee hemolymph has a variation in diet, age and hierarchy characteristics. (**B**) Metabolomics-based biomarker selection. Bee hemolymph extracts were processed by UHPLC-Orbitrap-MS for both untargeted and targeted metabolomics. In untargeted metabolomics, to assess the metabolic differences between the defined sample sets, PCA (principle component analysis) and OPLS-DA (orthogonal partial least square-discriminant analysis) were performed using Simca (soft independent modelling of class analogy)13 multivariate statistics software. Potential biomarkers annotation were consulted on online HMDB (Human Metabolome Database) and KEGG (Kyoto Encyclopedia of Genes and Genomes) database. In targeted metabolomics, Statistical analysis was performed with SPSS 22.0, applying two-way ANOVA and Tukey HSD test for post hoc comparisons to select significantly changed metabolites. To locate significantly expressed metabolites, the web-based platform MetaboAnalyst and KEGG database were used. Hereby, two candidate biomarker sets that could be associated with low-carbohydrate nutritional stress were selected from untargeted metabolomics (set 1) and targeted metabolomics (set 2), respectively. (**C**) Machine leaning algorithms are further implemented to build and evaluate the classification model. Classification sensitivity and specificity of the two selected biomarker sets (targeted and untargeted) and the combined set were tested using different machine learning algorithms. Well-validated classification models may be applied in real environments for bee health monitoring in terms of nutritional stress.

**Figure 2:**
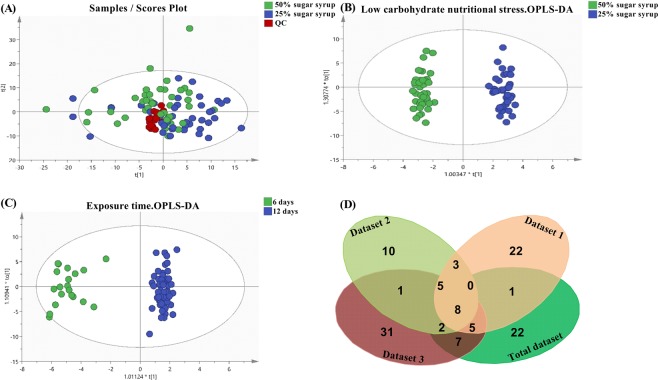
Plots from multivariate statistical analysis. (**A**) PCA-X score plots. Score plots for experimental (25% sugar syrup, n = 42), control (50% sugar syrup, n = 42), and quality control (QC) samples. (**B**) Low carbohydrate nutritional stress associated OPLS-DA score plot. (**C**) Exposure time associated OPLS-DA score plots. (**D**) Venn diagram of candidate biomarkers for nutritional stress from four datasets.

A schematic overview of the study design is presented in Fig. 1. A global data table characterizing each of the 84 samples by 2197 components was obtained and established as metabolomic fingerprints for *B*. *terrestris* hemolymph. PCA analysis revealed tight clustering of the QC samples (Fig. 2A), suggesting good instrumental stability during sample analysis. Datasets were validated by CV-ANOVA (*P* < 0.01) and permutation test. The OPLS-DA analysis showed a good discrimination in terms of nutritional stress (dataset T, Q^2^ = 0.616, R^2^Y = 0.981, Fig. 2B), even with variables from hierarchy and exposure time of the stressor. 6-day samples were also clearly distinct from 12-day samples (dataset T, Q^2^ = 0.754, R^2^Y = 0.958, Fig. 2C). By contrast, hierarchy effects on metabolic levels were not obvious (Table 1), although we observed dominance hierarchies which were established during the first days in queen-less conditions as previously described^26^. These results confirm that UHPLC-Orbitrap-MS-based metabolomics can successfully establish and distinguish the metabolic profiles of an individual *B*. *terrestris* using only microliter hemolymph samples.

**Table 1:** Classification dataset composition, and specification of constructed OPLS-DA models with output of model validation.

Dataset	Day 12 worker		Day 12 pseudo queen		Day 6 worker		Day 6 pseudo queen		Numbers of instances	Model specification	Numbers of model components (t_o_ + t_p_)^a^	Model characteristics^b^	Cross-validated ANOVA^c^	Permutation^d^
	25% syrup	50% syrup	25% syrup	50% syrup	25% syrup	50% syrup	25% syrup	50% syrup						
1	×	×							48	Diet	1 + 5 + 0	R^2^Y = 0.996 Q^2^ = 0.563	4.05 e^−7^	Good
2	×	×	×	×					64	Diet	1 + 6 + 0	R^2^Y = 0.998 Q^2^ = 0.656	0.001	Good
3	×	×			×	×			58	Diet	1 + 4 + 0	R^2^Y = 0.985 Q^2^ = 0.575	6.17 e^−6^	Good
T^e^	×	×	×	×	×	×	×	×	84	Diet	1 + 5 + 0	R^2^Y = 0.981 Q^2^ = 0.616	1.23 e^−10^	Good
4		×		×		×		×	42	Age	1 + 4 + 0	R^2^Y = 0.994 Q^2^ = 0.709	5.82 e^−6^	Good
5	×		×		×		×		42	Age	1 + 2 + 0	R^2^Y = 0.945 Q^2^ = 0.587	1.27 e^−5^	Good
T	×	×	×	×	×	×	×	×	84	Age	1 + 3 + 0	R^2^Y = 0.958 Q^2^ = 0.754	6.93 e^−20^	Good
6	×	×	×	×					64	Hierarchy	1 + 3 + 0	R^2^Y = 0.937 Q^2^ = 0.268	—	—
7					×	×	×	××	20	Hierarchy	1 + 0 + 0	R^2^Y = 0.798 Q^2^ = 0.599	—	—
T	×	×	×	×	×	×	×	×	84	Hierarchy	1 + 2 + 0	R^2^Y = 0.868 Q^2^ = 0.263	—	—

^a^with t_o_ the orthogonal and t_p_ the predictive component;

^b^with R^2^Y the variation in Y that is explained by the model, and Q^2^ the predictive ability of the model. Q^2^ > 0.5 indicated good model quality^58^;

^c^a cross-validated ANOVA *p* value < 0.05 indicated good model quality;

^d^good permutation testing was achieved if R^2^Y and Q^2^ values of the models based on the permutated data were significantly lower than those based on the real data set.

^e^total dataset.

Seven sub-datasets were further created containing specimens of different hierarchy and exposure time of the stressor (see Table 1). First, the results showed that the introduced low-carbohydrate stress had a striking effect on the hemolymph metabolome in all four analyzed datasets (dataset 1, 2, 3 and T), suggesting poor food availability disturbs bee metabolic states. This contributes to our understanding that sufficient food availability is vital for bee health. For bees, sugars are almost exclusively the substrates used for flight^27–29^, and they store only limited amounts of glycogen in the fat body and flight muscle tissues^30,31^. Hence, hemolymph energy supplies are very important for most activities. Second, similar performance of exposure time was observed both in control (dataset 4) and stressed datasets (dataset 5), indicating that age itself already had a clear metabolic effect. This suggests that the intrinsic physiological changes on *B*. *terrestris* hemolymph did not depend solely on extrinsic stressors, which is consistent with the assessment of honey bee senescence^32^. Third, the impact of hierarchy on the metabolic fingerprint is not obvious here (dataset 6, 7 and T). A possible explanation is that the dominance phenotype was not so clear-cut as expected. In the wild queenright colonies, multiple worker bees may also start laying drone eggs once the queen has started to produce new queens and drones^3^. Likewise, in microcolonies it has been observed, aside from the dominant worker, that also other bees tend to develop their ovaries (data not shown).

With more and more genomes of different bee species being published^33–37^, there is a growing number of comparative genomics, transcriptomics, and proteomics studies regarding bee health^38–42^. For most of these “omics” studies, the analysis has focused on explaining individual impacts or eventually emphasizing the complex nature of this problem. However, they have overlooked the methodological potential to disentangle complex interacting drivers^43^, and thus we still lack effective bee health monitoring and risk assessment tools. With respect to the current study, three following features may be noted when making the comparison with previous studies on bee stressors.

### Selection of diagnostic metabolic biomarkers for low-carbohydrate nutritional stress

**Figure 3:**
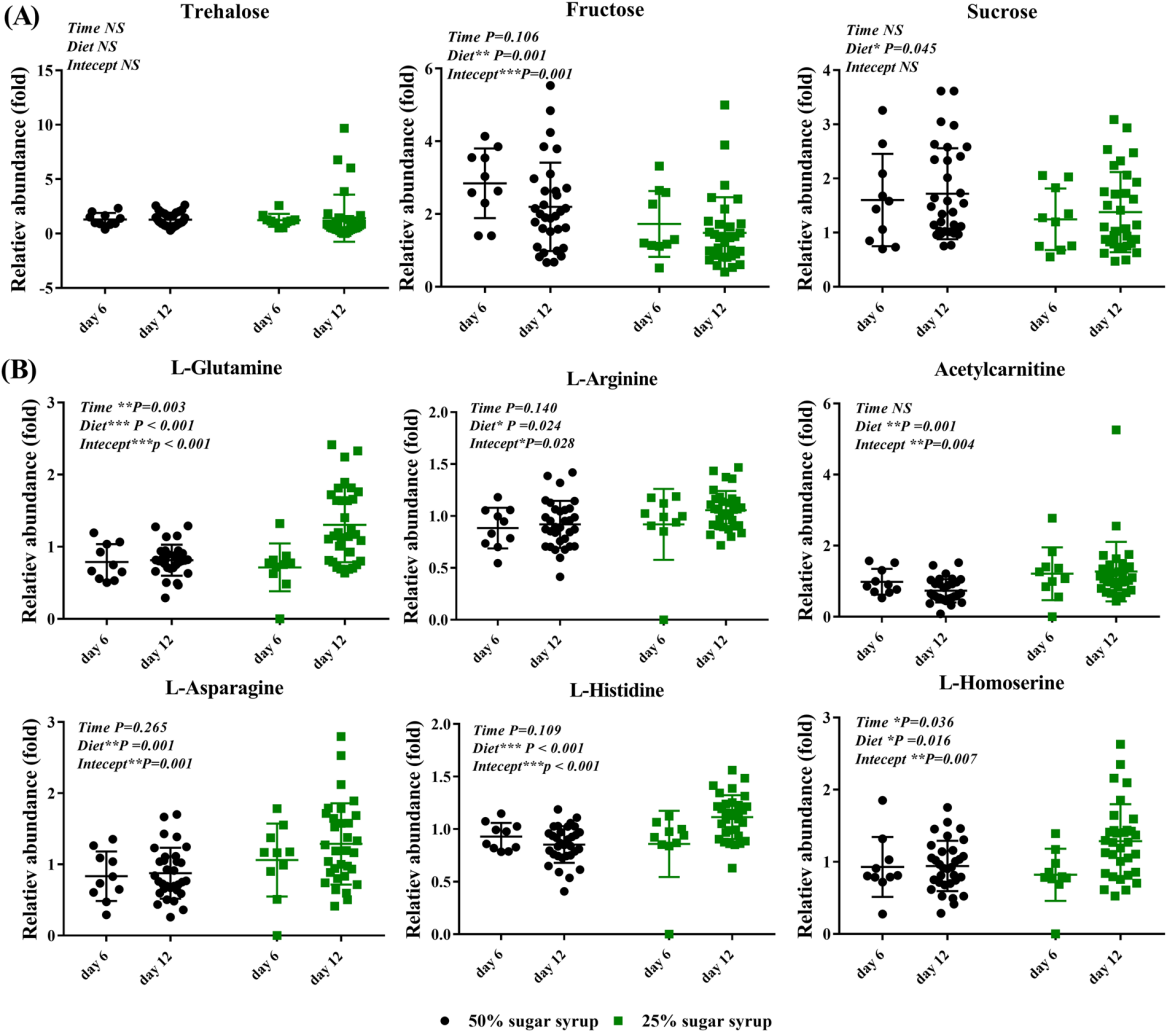
Comparison of nine key differential metabolites between diet (25% sugar syrup and 50% sugar syrup) and exposure time (day 6 and day 12). (**A**) main sugars. (**B**) main amino acids. The black cycles represent bees fed on 50% sugar syrup, and the green squares represent bees fed on 25% sugar syrup. Data were derived from targeted metabolomic assay data, and statistically significant results from two-way ANOVA comparing diet and age are indicated at the top left of the graphs. *P < 0.05, **P < 0.01, ***P < 0.001. NS = not significant.

A major feature of the current study is that we can promote biomarker discovery by selecting the candidate biomarkers not only through multiple validated OPLS-DA models but also by a dual targeted/untargeted data analysis strategy. As presented in Fig. 2D, with VIP > 1.0, a total of 44, 29, 59 and 45 components was assigned biomarker potential for nutritional stress in long-term stressed workers (dataset 1), long-term stressed bees (dataset 2), all stressed workers (dataset 3), and all stressed bees (total dataset), respectively. Furthermore, eight components were conserved in all four biomarkers sets and selected as the ideal untargeted biomarkers for nutritional stress. In parallel, a simultaneous targeted screening was performed. Among the nearly 300 metabolites included in our in-house library, 64 metabolites were identified and retained for semi-quantitative metabolic profiling. As summarized in Table 2, among these metabolites, there were 20% amino acids, 19% carbohydrates, 25% carboxylic acids and 36% of other chemical classes. Eight metabolites from sugar and amino acid metabolism were significantly changed during low-carbohydrate diet (Fig. 3) and selected as targeted biomarker set for nutritional stress. A total of 16 candidate biomarkers, including key metabolites of sugar and amino acid metabolism (set 2) and novel metabolites (set 1), are therefore ideal candidates to confirm our proof of concept.

**Table 2:** Peak abundance ratio of identified metabolites in bee hemolymph between 25% sugar syrup and control.

Metabolites	25% Sugar syrup/Control ratio on day 6		25% Sugar syrup/Control ratio on day 12	
	Pseudo queen	Worker	Pseudo queen	Worker
***Amino acids (20%)***				
Histidine* (His)	0.99	0.85	1.31	1.30
Arginine* (Arg)	0.95	1.17	1.08	1.21
Beta-Alanine(β-Ala)	0.53	0.90	1.00	1.00
Asparagine (Asn)	1.14	1.51	1.61	1.47
Aspartic acid (Asp)	0.67	1.85	1.23	0.74
D-Glutamic acid (Glu)	1.84	0.85	0.35	1.14
L-Glutamine (Gln)	0.76	1.13	1.85	1.55
Beta-Aminoisobutyric acid	0.61	1.22	1.41	1.28
Diaminopimelic acid	0.96	0.98	1.02	0.83
Hydroxyproline	0.60	1.07	1.51	1.05
Acetylcarnitine	0.95	1.64	3.03	1.45
Pyrrole-2-carboxylic acid	2.20	1.44	0.48	0.72
Homoserine	0.71	1.18	1.73	1.27
***Carbohydrates (19%)***				
Trehalose	0.92	0.95	0.58	1.21
Fructose	0.62	0.59	0.61	0.72
Sucrose	0.68	0.91	0.85	0.78
Rhamnose	0.45	0.81	0.71	0.95
N-Acetylneuraminic acid	0.74	0.82	0.50	1.38
D-Galacturonic acid	1.98	0.61	1.32	3.99
Gluconic acid	0.01	0.10	0.29	22.32
Sorbitol	1.44	1.31	0.83	0.64
Mannitol	0.88	0.49	0.52	0.46
Xylitol	0.72	5.96	7.96	12.46
***Carboxylic acid (25%)***				
Dihydrocaffeic acid	1.96	0.52	0.41	0.92
Pipecolic acid	1.11	0.82	1.16	1.33
3,4-Dihydroxybenzoic acid	2.48	0.69	0.61	0.43
3,4-Dihydroxyphenylacetic acid	3.41	1.06	0.26	0.90
4-Hydroxybenzoic acid	2.38	1.57	0.33	1.62
3-Hydroxybenzoic acid	1.17	1.08	0.90	1.01
Dihydroxy acid dehydratase	0.68	3.50	1.19	0.94
Vanillic acid	0.88	0.84	0.30	0.87
Homovanillic acid	0.55	0.74	0.61	0.82
3-Hydroxycinnamic acid	0.35	1.89	1.04	1.14
Phenylacetic acid	0.73	0.82	1.56	1.12
4-Methylvaleric acid	1.45	0.83	0.52	1.48
Hexanoic acid	1.38	0.56	0.45	1.25
Myristoleic acid	0.50	2.83	0.27	0.90
Vaccenic acid	3.67	5.31	4.43	1.37
***Various (36%)***				
D-Citramalate	1.93	1.20	0.04	0.34
α-Ketoglutaric acid	1.89	2.07	1.29	1.08
Phenylacetaldehyde	1.15	0.87	0.86	1.10
*Trans*-2-Octenal	0.63	0.36	0.84	1.40
3-Methyl-2-butenal	1.58	1.42	0.51	1.03
Gamma-Caprolactone	0.33	0.86	0.93	1.24
Methyl butyrate	0.53	0.60	0.92	1.14
Propyl acetate	0.90	0.78	1.01	1.05
Veratrole	0.39	0.65	1.12	1.50
2.4-Dimethyl-3-pentanone	0.41	0.45	1.78	0.90
3-Heptanone	0.36	0.44	1.76	0.88
2-Acetyl-5-methylfuran	0.15	0.20	0.31	0.40
Acetylpropionyl	1.42	0.45	1.56	1.10
3-Methyl-2-cyclohexen-1-one	0.87	0.82	0.75	1.15
Vanillylmandelic acid	0.94	0.55	0.25	0.77
4-Hexen-3-one	0.90	0.78	1.76	0.88
3-Phenyl-1-propanol	0.65	0.53	1.58	1.07
Styrene	1.25	1.18	0.84	0.82
Beta-Pinene	0.83	0.82	0.87	0.99
Hypoxanthine	0.32	0.20	1.33	1.02
2,6-Dimethylpyrazine	3.65	0.91	0.48	0.80
Tryptamine	1.40	0.88	0.67	1.30
Urocanic acid	0.61	0.87	0.58	0.69

### Application of machine learning algorithms to metabolomic data for stressor classification

A second distinctive feature is the application of machine learning algorithms on two selected biomarker sets to improve the sensitivity and accuracy of stressor diagnosis^44,45^. Within two selected biomarker sets, the further challenge is to validate the diagnostic performance of these selected biomarker sets. The evaluation of several well-known machine learning algorithms were therefore applied to four datasets separately. Results were summarized in Table 3, where sensitivity (recall) and specificity (precision) are displayed. The receiver operating characteristic (ROC) areas indicate a numerical value between 0 and 1 that describes the relationship between sensitivity and specificity for a given diagnostic test^46,47^. For the eight targeted key metabolites, good classification performance was concluded with ROC areas ranging between 0.64 and 0.951, indicating that our methodology was working well. Notably, combing the biomarkers sets from the targeted and untargeted approach resulted in ROC areas up to 0.99–1.0. This excellent performance can be mainly attributed to the biomarkers from the untargeted approach. With the growing evidence of nutritional stress as one major factor contributing to the synergistic effect of many other stressors and current bee colony declines^18–20,48^, this is the most clear test of the biomarker strategy concerning nutritional stress to date. Our results also suggest that combining metabolomics with data-driven machine learning algorithms has promising potential in evaluating bee health status and early risk assessment.

**Table 3:** Summary of the diagnostic accuracy (nutritional stress) of the machine learning algorithms analysis on four datasets.

Algorithm	Dataset	Targeted key metabolites			Untargeted biomarkers			Combined biomarker set		
		Specificity	Sensitivity	ROC area	Specificity	Sensitivity	ROC area	Specificity	Sensitivity	ROC area
**Random forest**	**1**	79.2%	79.2%	0.873	97.9%	98.0%	0.979	97.9%	98.0%	0.998
	**2**	78.1%	78.2%	0.870	96.9%	96.9%	0.999	96.9%	96.9%	0.999
	**3**	70.7%	70.7%	0.804	89.7%	90.4%	0.968	91.4%	91.8%	0.970
	**Total**	73.8%	73.8%	0.813	86.9%	88.0%	0.935	85.7%	86.5%	0.944
**LMT**	**1**	85.4%	85.5%	0.948	93.8%	93.8%	0.983	95.8%	96.2%	1
	**2**	85.4%	85.5%	0.930	95.3%	95.4%	0.988	96.9%	97.1%	1
	**3**	77.6%	77.6%	0.857	94.8%	95.3%	0.967	94.8%	95.3%	0.970
	**Total**	79.8%	79.9%	0.894	91.7%	92.9%	0.956	95.2%	95.7%	0.978
**J48**	**1**	79.2%	79.4%	0.796	97.9%	98.0%	0.979	97.9%	98.0%	0.979
	**2**	75.0%	75.0%	0.710	98.4%	98.5%	0.984	98.4%	98.5%	0.984
	**3**	70.7%	70.7%	0.673	93.1%	93.3%	0.943	93.1%	93.3%	0.943
	**Total**	64.3%	64.3%	0.671	85.7%	85.8%	0.898	84.5%	84.7%	0.845
**Logistic**	**1**	85.4%	85.5%	0.937	93.8%	94.4%	0.927	95.8%	96.2%	0.984
	**2**	81.3%	81.4%	0.891	96.9%	97.1%	0.973	96.9%	96.9%	0.999
	**3**	77.6%	77.9%	0.861	81.0%	81.4%	0.865	87.9%	91.4%	0.951
	**Total**	78.6%	78.6%	0.909	90.5%	90.6%	0.948	90.6%	90.6%	0.947
**Simple Logistic**	**1**	85.4%	85.5%	0.948	95.8%	96.2%	1	95.8%	96.2%	1
	**2**	84.4%	84.5%	0.930	96.9%	97.1%	1	96.9%	97.1%	0.999
	**3**	77.6%	77.6%	0.857	94.8%	95.3%	0.967	94.8%	95.3%	0.970
	**Total**	79.8%	79.9%	0.894	91.7%	92.9%	0.956	95.2%	95.7%	0.978
**Multilayer perceptron**	**1**	85.4%	85.5%	0.951	95.8%	96.2%	1	95.8%	96.2%	1
	**2**	85.9%	86.0%	0.943	96.9%	97.1%	1	100%	100%	1
	**3**	84.5%	84.5%	0.897	93.1%	93.9%	0.980	94.8%	95.3%	0.967
	**Total**	82.1%	82.2%	0.902	91.7%	92.3%	0.967	96.4%	96.7%	0.969
**NaiveBayes**	**1**	70.8%	71.0%	0.825	91.7%	91.7%	0.986	93.8%	93.8%	0.990
	**2**	75.0%	75.9%	0.838	92.2%	92.2%	0.971	93.8%	93.9%	0.975
	**3**	74.1%	75.6%	0.820	89.7%	89.7%	0.967	87.9%	88.0%	0.945
	**Total**	73.8%	75.9%	0.816	86.9%	87.1%	0.952	86.9%	87.4%	0.935
**BayesNet**	**1**	85.4%	85.5%	0.873	97.9%	98.0%	0.993	95.8%	96.2%	1
	**2**	75.0%	75.0%	0.761	96.9%	96.9%	0.988	98.4%	98.5%	0.998
	**3**	62.1%	62.1%	0.662	89.7%	88.3%	0.931	87.9%	88.0%	0.916
	**Total**	58.3%	58.3%	0.640	84.5%	85.5%	0.909	85.7%	86.5%	0.908

The normalized mass spectral ion intensities of the 8 key significantly expressed targeted metabolites and top 8 candidate biomarkers, marking food stress. All results were obtained using a 10-fold cross validation analysis.

### Biochemistry of low-carbohydrate nutritional stress

**Figure 4:**
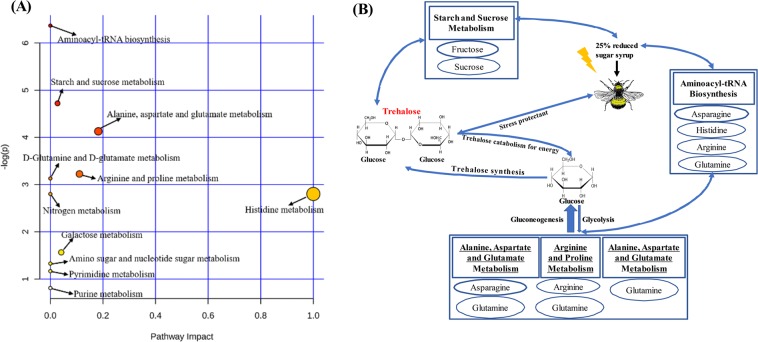
Pathway analysis of altered metabolites. (**A**) Summary of pathway analysis with MetaboAnalyst 3.0. Larger circles, higher and closer to the Y-axis, show a higher impact of the concerned pathway on the organism. (**B**) Scheme summarizing the proposed mechanisms underlying the response of bumblebee to low-carbohydrate food stress.

The third distinct feature is that we were able to investigate the underlying biochemistry mechanisms of low-carbohydrate stress, which can help to correlate overall results with the metabolites supervising both local and systemic response of bees to stressors^49^. Knowledge of the underlying biochemical pathway can help to identify additional markers perhaps not detected in the current approach. Under the introduced low-carbohydrate stress, a very characteristic decrease in metabolic profile regarding carbohydrates, and more abundant profiles of some compensating fatty acids and amino acids were observed. As could be expected, sucrose and fructose, two major sugars present in the supplied sugar syrup, were significantly decreased in experimental bees, although it was somewhat surprising that the blood sugar trehalose was extremely stable under low-carbohydrate stress (Fig. 3A). This decreased levels of carbohydrates is also observed in honeybees with energetic and nutritional stress imposed by *Nosema ceranae* infection^17,22^. Interestingly, the stable levels of trehalose was prolonged in control starved bees opposed to the infected ones^22^. Furthermore, the metabolism of several important amino acids, including histidine, arginine, asparagine, L-glutamine, acetylcarnitine, and homoserine, were positively impacted in bees fed with low-carbohydrate diet (Fig. 3B). Pathway analysis showed that several pathways were significantly disturbed including metabolism of amino acids, sugars, and nitrogen (Fig. 4A). Five pathways (aminoacyl-tRNA biosynthesis, alanine, aspartate and glutamate metabolism, arginine and proline metabolism, D-glutamine and D-glutamate metabolism, and starch and sucrose metabolism) with *P* value < 0.05 were considered to be significantly associated with low carbohydrate diet stress-induced metabolic changes. These down-regulated metabolites were only located into starch and sucrose metabolism, whereas, up-regulated metabolites were found in the other four metabolic pathways, including aminoacyl-tRNA biosynthesis, alanine, aspartate and glutamate metabolism, arginine and proline metabolism and D-glutamine and D-glutamate metabolism. To our knowledge, the obligatory precursor of protein synthesis is aminoacyl-tRNA (AA-tRNA)^50^, whereby aminoacyl-tRNA biosynthesis was the most disturbed pathway, suggesting an increased protein catabolism (denoted by a significant increase in glutamine) in those nutritionally stressed bees. These results provide evidence that nutritionally stressed bumblebees probably respond with increased protein catabolism, which has also been reported in *Diporeia*^51^.

These results encourage us to further explore the pathways linked to trehalose under nutritional stress in terms of metabolic homeostasis. Trehalose has been reported as the main energy source in insect hemolymph and as a stress protectant during extreme environmental conditions^52^. Since glutamine, the substrate of gluconeogenesis, was significantly increased (*P* < 0.0001) in hemolymph after feeding with low carbohydrate diet, we propose that gluconeogenesis may be significantly contributing to trehalose steady state in bumblebees with nutritional stress as shown in Fig. 4B. It is easy to understand that amino acids, the major fraction of the pollen, play a more important role in promoting responses to nutritional stress^53^. The supporting argument is that the endogenous amino acids are the main source utilized through gluconeogenesis, and the freshly ingested amino acids from pollen are to promote protein synthesis.

In conclusion, in this proof-of-concept study, we demonstrated that metabolomics-based methods, coupled with machine learning algorithms, represent valuable tools for the analysis of single bee stressor. In addition, this technique also shows power and potential as an assessment tool of bee health status in the real environment. The next stage should involve the identification of the untargeted biomarkers and development of a large cohort of wild sampling sites with various factors influencing bee health to test the categorizing accuracy of this approach for discovering biomarkers in multiple stressor risk assessment.

## Materials and Methods

### Study design

In this study, we aimed at retrieving and validating biomarkers for the introduced nutritional stress. It was considered important to have some physiological differences within our specimens, for which we had: (i) bees with different social hierarchy and age within the nest; (ii) bees facing different levels of malnutrition stress. The study was organized into three discrete phases as presented in Fig. 1: Phase A, the experiment setup, encompassing 2 × 8 microcolonies (control *vs* malnutrition), and bees were sampled at day 12. This experiment is regarded as “longer” exposure to stress. In each microcolony we sampled 3 workers and 1 dominant pseudo queen. Additionally, from 2 × 5 microcolonies we sampled one worker and one pseudo queen at day 6, where the stressor had less time to manifest. This experimental setup allowed us to create 8 datasets to investigate the effect of exposure time of the stressor, bee hierarchy, and their interaction, offering the ability to detect suitable metabolic markers related to nutritional stress. Table 1 represents the details of which groups of specimens were joined to create a specific dataset. Phase B, global hemolymph metabolic fingerprinting and profiling of samples from phase A, yielding two sets (from untargeted and targeted metabolomics) of potential biomarkers. Phase C, evaluation of the performance of the selected potential biomarkers using machine learning algorithms.

### Bumblebee microcolonies and mimic of malnutrition: low carbohydrate food forage

All experiments were performed using commercial *B*. *terrestris* callow workers obtained from Biobest (Westerlo, Belgium). The callow or newborn workers were randomly collected from small queenright colonies at their initial phase of start-up. Each worker originated from a different queen. Five random callow workers were distributed as one microcolony and all the microcolonies were randomly assigned to the experimental or control groups, a total of 26 microcolonies with 130 bumblebees was used in this study. These microcolonies were placed in an incubator at 30 °C, 60% relative humidity, continuous darkness, and were all fed with gamma-irradiated pollen (Apihurdes, Pinofranqueado, Spain). Control groups (13 microcolonies) received a standardized sugar syrup (50 w/v%, BIOGLUC, Biobest) consisting of sucrose, fructose, dextrose and maltose, while experimental groups (13 microcolonies) received a 25% sugar syrup to mimic low carbohydrate nutritional stress (diluted in distilled water).

### Hemolymph collection

Bee hemolymph was collected by making a small incision in the dorsal thorax and extracted for a total 10 μL per bee using Wiretrol II Capillary micropipettes (VWR) in phenylthiourea (PTU)-treated tubes to prevent melanization. The hemolymph sample was collected on ice and immediately put on dry ice afterwards. All hemolymph collection was performed under binocular microscope and three rules were strictly followed to guarantee the quality of sampling: i) the hemolymph should be pure and transparent; ii) no other tissues were perforated; iii) sampling time (incision and extraction) per bee is less than 35 seconds. All 84 samples were stored at −80 °C until chemical analysis.

### Generic extraction of polar metabolites from bee hemolymph

Since there are no protocols available for bumblebee hemolymph extraction of polar metabolites, two different solvent systems were tested in a preliminary experiment, i.e. methanol and methanol-ethyl acetate (v/v, 1/1), whereby the latter proved more efficient in achieving high metabolome coverage (35% higher with methanol-ethyl acetate). As such, 40 μL of methanol-ethyl acetate mixture was used for the extraction of polar metabolites. To remove proteins, all hemolymph samples were precipitated with extraction solvent, and 5 μL internal standard valine-d_8_ (ISTD, 25 ng/μL) was pre-added. Subsequently, samples were incubated at 4 °C for 30 min to enhance protein precipitation and centrifugated at 15,000 g for 15 min at 4 °C to remove the resulting precipitate. Ultimately, the supernatant was transferred to a 1.5 mL microfuge tube, and consecutively dried using the Speed-Vac. All dried samples were suspended in 100 μL ultrapure water and transferred to an LC-MS vial with glass insert. Solvents used for extraction of hemolymph metabolites were of LC-MS grade, and obtained from Fisher Scientific (Loughborough, UK) and VWR International (Merck, Darmstadt, Germany).

### UHPLC-Q-Orbitrap-HRMS analysis

The UHPLC-Orbitrap-MS method that was used in this study was adopted from^54^ as previously optimized^55^. An external standard mixture containing ca. 300 metabolites (including amino acids, monocarboxylic acids, phenols, multi-carboxylic acids, amines, carbohydrates, polyols, short chain fatty acids, inorganic acids, bile salts, and N-compounds) was used to assess instrumental performance and execute targeted profiling. A pool of all extracts (n = 84) was used to make quality control (QC) samples for instrument conditioning (external QC samples) and data normalization (internal QC samples). The Q-Exactive^TM^ Orbitrap mass analyzer (Thermo Fisher Scientific, San Jose, CA) was equipped with a heated electrospray ionization (HESI II) operating in polarity switching mode. An Acquity UPLC HSS T3 column (1.8 μm, 150 mm × 2.1 mm) (Waters, Zellik, Belgium) was used, whereby a binary solvent system using ultrapure water (A) and acetonitrile (B), both acidified with 0.1% formic acid, was applied at a constant flow rate of 0.4 mL min^−1^. All Solvents used were of LC-MS grade. Experimental samples were run in a randomized order (except for quality control samples, which were analyzed in duplicate after every nine experimental samples).

### Untargeted data analysis and metabolic fingerprinting construction

The LC–MS raw data were first exported using Xcalibur^TM^ 2.1 (Thermo Fisher Scientific, CA) and imported into Compound Discoverer 3.0 software (Thermo Fisher Scientific, CA) with the untargeted metabolomics workflow and differential analysis mode. As major parameters, a minimum peak intensity of 500,000 a.u., retention time width of 0.25 min, *m*/*z* scan range from 53.4–800 dalton, and *m*/*z* width of 6 ppm were applied for feature extraction. The final data matrix was composed of the peak intensities for the detected components (rows) and different samples (columns), and was exported to an excel file. The coefficient of variation (%CV) was calculated for each component in the collection of QC samples, and components with %CV lower than 30%, which is considered an acceptable value of repeatability in untargeted metabolomics^56^, were retained. Data normalization was then performed by dividing the peak intensity of each metabolite in every sample by its corresponding mean peak intensity, as determined based on the following two internal QC samples^54^. To assess the metabolic differences between the defined samples sets, PCA (principal component analysis) and OPLS-DA (orthogonal partial least square-discriminant analysis) were performed in Simca^TM^ 13 (Umetrics, Malmo, Sweden). S-plots were built using validated OPLS-DA models in order to select metabolites that are important for classifying treatment (full data set) and different levels under treatment (day 6 and day 12 data set). A variable importance in projection (VIP) plot was applied to evaluate the importance of a certain components with VIP-value > 1.0.

### Targeted data analysis and putative metabolic mechanisms underlying low-carbohydrate stress

Xcalibur^TM^ 2.1 (Thermo Fisher Scientific, San Jose, USA) was used to process the data (peak area determination) from metabolites that were identified based on the *m*/*z* and retention time from those metabolites that were included in the in-house database (±300 compounds). Statistical analysis was performed using SPSS 22.0 software, applying two-way ANOVA and Tukey HSD test for post hoc comparisons, whereby a *P* < 0.05 was considered as statistically significant. To locate those significantly expressed metabolites, the metabolic pathways were drawn based on the knowledge of those metabolites and the web-based platform MetaboAnalyst (http://www.metaboanalyst.ca/). Investigation of hemolymph molecules by targeted analysis is envisaged to correlate overall results including those from the untargeted fingerprinting and provide more biochemical information, hence, the underlying metabolic mechanisms of bees’ response to nutritional stress was further explored.

### Machine learning algorithms implementation

To provide a quantitative diagnostic approach for adaption to a field-based monitoring, we need tools capable of extending the utility of the selected biomarker sets from a complex multivariate analysis to an applicable binary or categorizable format. Within this context, machine learning methods along with several more specific classification algorithms were tested to reveal the categorical accuracy of the candidate biomarker signatures. Standard implementations of the classification algorithms were performed with the Waikato Environment for Knowledge Acquisition (WEKA, University of Waikato, New Zealand, https://www.cs.waikato.ac.nz/ml/weka/)^57^ with 10-fold cross-validation settings.

## Data Availability

The datasets generated and analyzed during the current study are available from the corresponding author on reasonable request.
